# Clinical Assessment and Factors Influencing Treatment Strategy and Outcomes in Complex Limb Injuries

**DOI:** 10.7759/cureus.73347

**Published:** 2024-11-09

**Authors:** Liam Donnelly, Henry J Mills

**Affiliations:** 1 Trauma and Orthopaedics, Barts Health NHS Trust, London, GBR; 2 Orthopaedics, University Hospital Geelong, Geelong, AUS

**Keywords:** complex limb injuries, crush injuries, damage control orthopaedics, injury severity scores (iss), limb reconstruction surgery, limb-salvage, major limb amputation, mangled extremity severity score, mangled limb, orthoplastic surgery

## Abstract

Complex limb injuries are high-energy injuries that may threaten limb viability and confer significant morbidity and mortality. Prompt clinical assessment and decision-making are critical to determining the optimal management strategy, i.e., limb salvation or amputation. A clinical assessment based on advanced trauma life support (ATLS) principles is conducted to identify injuries that threaten life or limb. Patients' physiological status is evaluated using validated scoring tools, such as the injury severity score, to identify high-risk individuals who may need treatment following damage control orthopedic principles. Clinicians must assume a high degree of suspicion of vascular injury and may reliably use CT angiography to detect clinically relevant vascular injuries. Several clinical tools have been developed to help decide between primary amputation or salvation of mangled limbs; however, none have proven valid as sole indicators. Decision-making must involve the consideration of a multitude of injury- and patient-related variables in addition to clinical scoring systems. This review provides an outline of the clinical assessment and factors influencing treatment strategy and outcomes in complex limb injuries based on current evidence.

## Introduction and background

Limb trauma represents one of the most common injury patterns seen in emergency medical and surgical practice, with 1.8 million fractures occurring each year in England alone [1,2]. Complex limb injuries (CLI), which involve severe damage to the anatomical components of extremities: soft tissue, bone, vasculature, and nerves, account for only a minority of these cases but are associated with significant patient morbidity and healthcare resource burden [2]. The term ‘mangled limb’ typically refers to the most devastating injuries, which involve three or more soft tissue, bone, vasculature, and nerve structures [1,3,4]. CLI typically results from high-energy mechanisms, predominantly penetrating or combined injuries in military settings, and road traffic accidents and industrial/farm accidents in civilian populations [1,5]. Such injuries raise concern for the integrity and viability of the injured limb or may be life-threatening in severe cases and are associated with considerable early and long-term morbidity [5].

A timely and accurate clinical assessment is crucial to identify concomitant life-threatening injuries and evaluate the viability of the affected limb, thereby guiding treatment strategy. Surgical management of CLI comprises two options: limb salvage or amputation [6]. Historically, the majority of CLI were treated with primary amputation [3]. However, advancements in microsurgical techniques and integrated trauma care systems have increased the feasibility of limb salvage [7]. Despite these advances, no consensus or validated scoring tool exists to guide treatment choices definitively. Consequently, decision-making poses a significant challenge to the trauma team when managing patients with CLI.

This review aims to provide an outline of the key aspects of assessment and treatment strategies, focusing on initial evaluation, vascular injury assessment, and the factors influencing limb salvage versus amputation based on current evidence.

## Review

Initial assessment

Primary Survey

A comprehensive primary survey according to advanced trauma life support (ATLS) principles should be performed in the first instance [3,7]. Prompt identification and treatment of life-threatening injuries are crucial in CLI patients, who are usually poly-traumatized due to high-energy mechanisms but may present with distracting extremity injuries [4]. Catastrophic bleeding from an extremity should be prioritized above the assessment and management of airway, breathing, and circulation (ABC) [8]. Studies demonstrate that catastrophic hemorrhage represents the leading cause of preventable death in trauma, with extremity wounds causing a significant number of deaths in military settings [8]. Accordingly, a systematic approach to the initial assessment is paramount in patients presenting with CLI. In case of catastrophic extremity bleeding, hemorrhage control can be quickly obtained by direct pressure with escalation to tourniquet as per British Orthopaedic Association Standards for Trauma and Orthopaedics (BOAST) guidelines, with simultaneous initiation of the massive hemorrhage protocol [7,9].

Effective pain relief is important during the initial assessment of trauma patients in the emergency department; inadequately treated pain can contribute to poor patient outcomes [10]. National Institute for Health and Care Excellence (NICE) guidelines recommend intravenous morphine as the first-line analgesic for patients with major trauma [11]. The use of intravenous ketamine, which is the recommended second-line analgesic by NICE, avoids opiate-related adverse effects and has demonstrated effective pain relief in a recent study involving patients with long bone fractures [10].

Physiological Assessment

Evaluation of patient’s physiological status and injury burden during the initial assessment is critically important to guide resuscitation and acute treatment strategies. MacKenzie and Bosse [12] report that 30% of trauma surgeons consider patients’ overall Injury Severity Score (ISS) as the single most important factor when making the decision to amputate versus salvage [7]. Moreover, patients with CLI may be vulnerable to the phenomenon known as the ‘second hit,’ which describes the additional physiological insult caused by surgical intervention in polytraumatized patients [13]. The phenomenon dictates that patients who have suffered severe physiological insults are at high risk of suffering post-operative systemic complications [14]. High-risk patients who present with life-threatening injuries are often managed according to damage control orthopaedic (DCO) principles, which prioritizes physiological restitution over anatomic fixation, in contrast to early definitive fixation, which typically invokes a greater ‘second hit’ [14-16]. DCO aims to minimize the surgical duration and bleeding and achieve early rapid fracture stabilization through temporizing measures, such as external fixation, fasciotomy, and vascular shunting [16]. Pape et al. describe a four-tier classification (stable, borderline, unstable, and in extremis) that stratifies patients according to their injury type and physiological and biochemical parameters [17]. Authors report that DCO yields more favorable outcomes for patients who were classified as unstable or in extremis, compared to early definitive fixation [17]. Notwithstanding, patients who have been adequately resuscitated should undergo early definitive fixations within 36 hours to avoid complications associated with delayed fixation, as per Vallier et al.’s [16] ‘Early Appropriate Care’ protocol.

Assessment of Vascular Injury

Clinicians should assume a high degree of suspicion of vascular injury in CLI, especially in cases of supracondylar femur fractures, knee dislocations, proximal tibia fractures, and penetrating injuries of the posterior and medial thigh [3]. In 1992, Frykberg categorized ‘hard’ and ‘soft’ clinical signs of vascular injury to inform treatment decision-making [18]. ‘Hard’ signs, which include an absent distal pulse, distal ischemia, active hemorrhage, large hematoma, bruit, or thrill, indicate the need for immediate surgical exploration without preoperative angiography [14]. Conversely, ‘soft’ signs (history of hemorrhage, small hematoma, hypotension, or deficit of associated peripheral nerve) were not considered to be predictive of arterial injury requiring surgical intervention [18]. Despite significant advances in trauma care since their inception, the dogma of ‘hard’ and ‘soft’ signs of vascular injury remains the foundation of bedside vascular assessment of an injured limb [19].

Over recent years, however, the management of patients exhibiting ‘soft’ signs has changed somewhat. Firstly, the Ankle Brachial Pressure Index (APBI) superseded conventional arteriography (CA), with studies reporting that an ABPI ≥0.9 reliably excludes arterial injury and thus prevents unwarranted exploration [19-21]. More recently, Seamon et al. proposed that CT angiography (CTA) replace CA as the gold-standard imaging modality for extremity vascular injuries, reporting that CTA detected clinically relevant vascular injuries with 100% sensitivity and specificity [22]. Furthermore, a 2021 report by Romagnoli et al. dissuades the use of outdated ‘hard’ and ‘soft’ signs given the modern ubiquitous availability of CTA following a review of the American Association for the Surgery of Trauma PROspective Observational Vascular Injury Treatment (PROOVIT)registry data [19]. Specifically, Romagnoli et al.argue that the presence of ‘hard’ signs does not correlate with physiological derangement (based on ISS and physiological parameters), nor does it bear predictive utility regarding the necessity of operative intervention. Instead, authors advocate that hemorrhagic and ischaemic signs of vascular injury offer far greater clinical utility in guiding imaging and treatment [19].

Decision-making, limb salvage, or amputation

The decision to salvage a limb or proceed with primary amputation is complex, requiring consideration of multiple patient and injury factors. Limb salvage represents an arduous treatment path for patients, who must endure prolonged rehabilitation, several surgical procedures, and higher rates of complication and rehospitalization compared to amputees [14,23]. On the other hand, primary amputation is a highly emotive and irreversible life-altering decision [6]. Several clinical tools have been developed to aid decision-making, and more recently, machine-learning modules have shown promise in predicting failed limb salvage [24]. However, clinical judgment remains paramount in decision-making due to a lack of both consensus and high-quality evidence [1,6]. Moreover, the literature outlines that equivalent functional outcomes are achievable with both limb salvage and amputation, suggesting that an individualized approach to decision-making is preferable [14]. Consequently, a multidisciplinary team involving trauma, orthopaedic, vascular, and plastic surgeons must carefully consider the clinical condition of the limb, patient comorbidities, patient preference, surgical expertise as well as utilizing tools when deciding to salvage or amputate [14].

Scoring Systems

Introduced by Johansen in 1990, the Mangled Extremity Severity Score (MESS) considered the extent of skeletal and soft tissue injury, limb ischemia, shock, and patient age to predict the need for amputation after limb injury [25]. Authors report that a MESS score ≥7 predicted amputation with 100% accuracy in their study of 51 patients [25]. Despite its widespread utilization over subsequent years, MESS has been subject to growing concerns over its prognostic accuracy following advances in the management of CLI, including the increased use of tourniquets and improvements in reconstructive surgery [4,26]. Studies have not been able to corroborate the 100% accuracy initially reported by Johansen et al. [3,26]. Notably, a 2015 systematic review by Schiro et al. reports a wide range of accuracy for a MESS score ≥7 (0-93.4%), casting further doubt over the unreliability of MESS [27]. In response to criticisms, McNamara et al. developed the score for nerve injury, ischemia, soft-tissue injury, skeletal Injury, shock, and age of patient (NISSSA), a modification of the Mangled extremity severity score (MESS), which includes a nerve injury component [28]. The authors concluded that NISSSA was more sensitive and specific than the MESS following a small retrospective study of 24 patients with type IIIB and type IIIC (Gustilo and Anderson) open tibia fractures [28]. Notwithstanding, the NISSSA has been criticized for placing too much emphasis on the loss of plantarflexion in the acute phase, as this is often a neuropraxia that resolves spontaneously [3,29].

Alternative scoring systems were developed in an attempt to more reliably predict primary amputation, including the limb salvage index (LSI), predictive salvage index (PSI) and the Hannover fracture scale (HFS) [26]. The systems consider factors such as patient age, presence of shock, neuro vasculature and musculotendinous damage, degree of contamination, warm ischemia, time, and time to treatment [26]. In 2001, Bosse et al. prospectively evaluated the five aforementioned scoring systems in a multicentre project involving 556 patients with high-energy lower extremity injuries (lower extremity assessment project (LEAP)) [29]. Analysis failed to validate the clinical utility of any of the scoring systems for predicting the need for amputation [29]. In addition to their lack of validity, these scoring systems consider neither the availability of personnel and resources nor subjective patient-related factors which have been shown to influence outcomes, such as pre-injury functional baseline, psychological and socioeconomic status, occupation, and available support system [12,23,30]. Therefore, clinicians should adopt a holistic approach rather than relying on scoring systems as a sole criterion when deciding between limb salvage and amputation.

Independent Indicators for Amputation

Evidence suggests that factors influencing the decision to salvage or amputate should be considered as relative criteria, not absolute indicators for amputation [14]. The exception to this is when life-saving primary amputation is necessitated to urgently limit blood loss and avoid prolonged operating time in patients presenting with higher-energy mechanisms, significant shock, or concomitant severe head injury [5]. Debate surrounds even the most influential factors, such as limbs with transected nerves (particularly sciatic and tibial nerves) unamenable to reconstruction and crushed extremities with an ischemia time greater than six hours [4]. For example, a 2021 expert committee proposed that the six-hour ischemia time limit has only a relative significance [14]. This was based on a meta-analysis by Fowler et al., which concluded that long ischemia time was not predictive of amputation and instead cited studies that suggested amputation was more likely to be due to soft tissue issues than ischemia [31]. Indeed, soft tissue loss is widely considered as one of the most influential factors for primary amputation, however it must be noted that advances in microsurgical and tissue coverage capabilities have contributed to an increase in limb salvage attempts [3,14,26].

Consideration of Comorbidities and Complication Risk

Several authors recommend that both surgeon and patient must understand the expected complication risk when deciding to salvage or amputate [3,32]. An analysis of 545 patients involved in the LEAP by Harris et al. emphasizes the significant complication rates following both procedures, particularly limb salvage [32]. Overall, the two most common complications were wound infection (28.3%) and nonunion (23.7%) [32]. Limb salvage patients were particularly prone to nonunion (31.3%), wound infection (23.2%), and osteomyelitis (8.6%) [32]. In contrast, amputees suffered wound infection (34.2%), wound breakdown (13.4%), and phantom limb pain (13.4%) [32]. The complication risk for each patient will vary depending on patient-related variables; for example, smoking and diabetes mellitus are well-documented predisposing factors for infection and nonunion [32]. Castillo et al. report that smokers are 37% less likely to achieve union, twice as likely to develop an infection, and 3.7 times as likely to develop osteomyelitis compared to non-smokers [33]. Overall, it is widely agreed that comorbidities represent an important consideration when deciding to salvage or amputate, thus reiterating the highly individualized nature of this decision [4].

## Conclusions

Limb salvage capabilities have dramatically improved in recent years due to advances in trauma care, including microsurgical and tissue coverage techniques. However, the decision to salvage or amputate in complex limb injuries remains contentious. Multiple scoring systems have been developed with the intention of objectifying the decision to amputate. However, none have proven to be valid. Instead, decision-making should be governed by the careful consideration of a multitude of injury and patient-related variables; scoring tools, while helpful, should not be the sole basis for treatment decisions. Most patients will not have an absolute indication for amputation; therefore, the surgeon and patient must examine the likely implications and outcomes of both procedures to determine an individually tailored treatment plan. Future research should focus on developing more accurate and patient-centered decision-making tools.

## References

[1] Prasarn ML, Helfet DL, Kloen P (2012). Management of the mangled extremity. Strategies Trauma Limb Reconstr.

[2] (2024). Fractures (complex): assessment and management. https://www.nice.org.uk/guidance/ng37.

[3] Bumbaširević M, Matić S, Palibrk T, Glišović Jovanović I, Mitković M, Lesić A (2021). Mangled extremity-modern concepts in treatment. Injury.

[4] (2024). Severe lower extremity injury in the adult patient. https://www.uptodate.com/contents/severe-lower-extremity-injury-in-the-adult-patient.

[5] de Mestral C, Sharma S, Haas B, Gomez D, Nathens AB (2013). A contemporary analysis of the management of the mangled lower extremity. J Trauma Acute Care Surg.

[6] Al-Hourani K, Pearce O, Kelly M (2021). Standards of open lower limb fracture care in the United Kingdom. Injury.

[7] Sheffield AC, Barton D, Ebler DJ, Morandi MM, Skarupa DJ (2023). Management of the mangled extremity. Curr Surg Rep.

[8] Hodgetts TJ, Mahoney PF, Russell MQ, Byers M (2006). ABC to ABC: redefining the military trauma paradigm. Emerg Med J.

[9] (2024). Diagnosis & management of arterial injuries associated with extremity fractures and dislocations. https://www.boa.ac.uk/resource/boast-6-pdf.html.

[10] Yılmaz M, Kudu E, Sanri E, Karacabey S, Akoglu H, Denizbasi A (2023). Comparison of the analgesic effects of low-dose ketamine versus fentanyl in patients with long bone fractures in the emergency department: a prospective observational study. Cureus.

[11] (2024). Major trauma: assessment and initial management. https://www.nice.org.uk/guidance/ng39.

[12] MacKenzie EJ, Bosse MJ (2006). Factors influencing outcome following limb-threatening lower limb trauma: lessons learned from the Lower Extremity Assessment Project (LEAP). J Am Acad Orthop Surg.

[13] Pape HC, Halvachizadeh S, Leenen L, Velmahos GD, Buckley R, Giannoudis PV (2019). Timing of major fracture care in polytrauma patients: an update on principles, parameters and strategies for 2020. Injury.

[14] Pottecher J, Lefort H, Adam P (2021). Guidelines for the acute care of severe limb trauma patients. Anaesth Crit Care Pain Med.

[15] Devendra A, Nishith PG, Dilipchand RS, Dheenadhayalan J, Rajasekaran S (2021). Current updates in management of extremity injuries in polytrauma. J Clin Orthop Trauma.

[16] Vallier HA, Moore TA, Como JJ (2015). Complications are reduced with a protocol to standardize timing of fixation based on response to resuscitation. J Orthop Surg Res.

[17] Pape HC (2008). Effects of changing strategies of fracture fixation on immunologic changes and systemic complications after multiple trauma: damage control orthopedic surgery. J Orthop Res.

[18] Frykberg ER (1992). Arteriography of the injured extremity: are we in proximity to an answer?. J Trauma.

[19] Romagnoli AN, DuBose J, Dua A (2021). Hard signs gone soft: a critical evaluation of presenting signs of extremity vascular injury. J Trauma Acute Care Surg.

[20] Feliciano DV (2017). Pitfalls in the management of peripheral vascular injuries. Trauma Surg Acute Care Open.

[21] Spitler CA, Patch DA, McFarland GE, Smith WR (2022). Assessment and interventions for vascular injuries associated with fractures. J Am Acad Orthop Surg.

[22] Seamon MJ, Smoger D, Torres DM (2009). A prospective validation of a current practice: the detection of extremity vascular injury with CT angiography. J Trauma.

[23] Busse JW, Jacobs CL, Swiontkowski MF, Bosse MJ, Bhandari M (2007). Complex limb salvage or early amputation for severe lower-limb injury: a meta-analysis of observational studies. J Orthop Trauma.

[24] Perkins ZB, Yet B, Sharrock A, Rickard R, Marsh W, Rasmussen TE, Tai NR (2020). Predicting the outcome of limb revascularization in patients with lower-extremity arterial trauma: development and external validation of a supervised machine-learning algorithm to support surgical decisions. Ann Surg.

[25] Johansen K, Daines M, Howey T, Helfet D, Hansen ST Jr (1990). Objective criteria accurately predict amputation following lower extremity trauma. J Trauma.

[26] Loja MN, Sammann A, DuBose J (2017). The mangled extremity score and amputation: time for a revision. J Trauma Acute Care Surg.

[27] Schirò GR, Sessa S, Piccioli A, Maccauro G (2015). Primary amputation vs limb salvage in mangled extremity: a systematic review of the current scoring system. BMC Musculoskelet Disord.

[28] McNamara MG, Heckman JD, Corley FG (1994). Severe open fractures of the lower extremity: a retrospective evaluation of the Mangled Extremity Severity Score (MESS). J Orthop Trauma.

[29] Bosse MJ, MacKenzie EJ, Kellam JF (2001). A prospective evaluation of the clinical utility of the lower-extremity injury-severity scores. J Bone Joint Surg Am.

[30] McLaughlin CM, McLaughlin CJ, Candela X, Parham CS, Roberts JM (2022). Predictive characteristics of limb salvage versus amputation in lower extremity trauma: a review of the national trauma data bank. Ortho Surg.

[31] Fowler J, Macintyre N, Rehman S, Gaughan JP, Leslie S (2009). The importance of surgical sequence in the treatment of lower extremity injuries with concomitant vascular injury: A meta-analysis. Injury.

[32] Harris AM, Althausen PL, Kellam J, Bosse MJ, Castillo R (2009). Complications following limb-threatening lower extremity trauma. J Orthop Trauma.

[33] Castillo RC, Bosse MJ, MacKenzie EJ, Patterson BM (2005). Impact of smoking on fracture healing and risk of complications in limb-threatening open tibia fractures. J Orthop Trauma.

